# Social inequity in chiropractic utilisation – a cross-sectional study in Denmark, 2010 and 2017

**DOI:** 10.1186/s12998-024-00548-x

**Published:** 2024-07-15

**Authors:** Kristine Bihrmann, Michelle Trabjerg Pedersen, Jan Hartvigsen, Kirstine Wodschow, Annette Kjær Ersbøll

**Affiliations:** 1 National Institute of Public Health, University of Southern Denmark, Studiestraede 6, 1455 Copenhagen, Denmark; 2 Center for Muscle and Joint Health, Department of Sports Science and Clinical Biomechanics, University of Southern Denmark, Odense, Denmark; 3 Chiropractic Knowledge Hub, University of Southern Denmark, Odense, Denmark

**Keywords:** Chiropractic utilisation, Inequity, Education, Income, Employment, Health disparities

## Abstract

**Background:**

Inequity in healthcare utilisation refers to differences between groups that remain after adjustment for need for health care. To our knowledge, no previous studies have aimed to assess social inequity in chiropractic utilisation in a general population. Therefore, the objective of this study was to evaluate social inequity in chiropractic utilisation in the general Danish population adjusted for health status as a proxy of need for chiropractic care.

**Methods:**

A population-based repeated cross-sectional study design was used based on the Danish National Health Survey in 2010 and 2017. Overall, we included 288,099 individuals aged 30 years or older in 2010 or 2017. For each individual, information on chiropractic utilisation, socioeconomic status, and health status as a proxy of need for chiropractic care was retrieved from nationwide registers using the unique personal identification number. Measures of health status included demographics, poor self-rated physical health, activity limitations, musculoskeletal pain, number of musculoskeletal conditions, and number of chronic diseases.

We investigated social inequity in chiropractic utilisation (yes, no) using logistic regression adjusted for health status, stratified by sex and year. Three characteristics of socioeconomic status (educational level, employment status and income) were investigated. To further quantify the degree of social inequity in chiropractic utilisation, we estimated the concentration index of inequity for each of the three characteristics of socioeconomic status.

**Results:**

We found significantly higher odds of chiropractic utilisation among individuals with short or medium/long education compared with individuals with elementary education, and among employed individuals compared with individuals who were unemployed, receiving disability pension or retired. Furthermore, the odds of chiropractic utilisation increased with higher income. The concentration index indicated social inequity in chiropractic utilisation in favour of individuals with higher socioeconomic status, with income and employment status contributing more to inequity than educational level.

**Conclusion:**

The study demonstrated social inequity in chiropractic utilisation in Denmark beyond differences in health status as a proxy of need for chiropractic care in the general population. The results suggest that new strategies are required if equal treatment for equal need is the goal.

**Supplementary Information:**

The online version contains supplementary material available at 10.1186/s12998-024-00548-x.

## Introduction

Chiropractic is defined as a health profession concerned with the diagnosis, treatment, and prevention of mechanical disorders of the musculoskeletal system, and the effects of these disorders on the function of the nervous system and general health [1]. Globally, chiropractors are present in 90 out of 193 United Nation member countries where they primarily function in private clinics. More than half of the countries fully or partially cover services provided by chiropractors [2]. Chiropractors use a range of therapies in their practice with spinal manipulation as the most common treatment provided [3, 4]. In the general population, back pain is the most common reason for seeking chiropractic care [3, 5, 6].

In Denmark, chiropractic is a well-established and integrated part of the healthcare system. Services are directly available without referral from general practitioners, but unlike a wide range of other health services in Denmark, chiropractic care is not free of charge. In fact, out-of-pocket expense for patients constitutes around 80% of the fee [7]. In 2017, out of a total of almost six million Danes, almost two million had a private health insurance that often fully or partially cover chiropractic services [8]. The vast majority were insured as part of a job agreement [9]. Around 2 million Danes are members (self-paid) of a non-profit health insurance organisation which also reimburses part of the co-payment [10]. In 2010, the Danish population was served by 326 chiropractors practicing in private clinics [11]. By 2017, this number had increased to 416.

Internationally, a median of 9.1% of the general population utilise chiropractic services during a 12-month period [3]. In Denmark, 6.6% consulted a chiropractor in 2022 [12]. Most studies on chiropractic utilisation have been conducted in United States, Canada, or Australia, where some differences in chiropractic utilisation between socioeconomic groups have been found [13].

When evaluating differences in healthcare utilisation between population groups it is important to account for differences in need for health care between groups. Otherwise, results may reflect differences in need between groups rather than relevant differences in utilisation. Consequently, inequity in healthcare utilisation refers to differences between groups that remain after adjustment for need for health care, with health status being a commonly used proxy of need [14, 15].

There is no standardised way to assess need for chiropractic care in a population group, but review findings have shown that musculoskeletal conditions are by far the main reason for seeking chiropractic care [3]. Since the prevalence and impact of musculoskeletal conditions vary between socioeconomic groups [16, 17], it is of great importance to account for this when evaluating differences in chiropractic utilisation between socioeconomic groups. To our knowledge, however, no previous studies have aimed to assess social inequity in chiropractic utilisation in a general population.

The objective of this study was to evaluate social inequity in chiropractic utilisation in the general Danish population adjusted for health status as a proxy of need for chiropractic care. Furthermore, the development in inequity over time was evaluated. Three different characteristics of socioeconomic status were used: educational level, employment status, and income.

## Materials and methods

### Study design and population

A population-based repeated cross-sectional study design was used based on the Danish National Health Survey (DNHS) in 2010 and 2017 [18]. In brief, the survey is a large population-based survey with a representative sample of the Danish adult population (≥ 180.000 participants, age 16 years or older). The overall aim of the DNHS is to monitor status and trends in physical and mental health, health behaviour, and morbidity [18]. The survey is repeated every 3–4 years. The study design, data collection procedure and response rates of the surveys are described in more detail elsewhere [18].

Two separate study populations were established based on individuals participating in the DNHS in 2010 and 2017, respectively. For each individual in the study populations, information on chiropractic utilisation, socioeconomic status, and health status was retrieved from nationwide Danish registers using the unique personal identification number that is given to all Danish residents at birth or immigration [19].

Individuals were excluded from the study if they were younger than 30 years in 2010 and 2017, respectively, and in case of missing information on variables characterising socioeconomic status or health status. The study population was limited to individuals of at least 30 years of age as they are expected to have reached their final educational level.

### Chiropractic utilisation

Chiropractic utilisation was defined as a binary variable indicating whether an individual had at least one chiropractic consultation during a specific year. All chiropractic consultations are registered in the Danish National Health Service Register (specialty codes 53 and 64), which is a nationwide register with information about activities of health professionals contracted with the tax-funded primary public healthcare system [20]. Chiropractic utilisation was assessed in 2010 and 2017, respectively.

### Socioeconomic status

Socioeconomic status was assessed using three different characteristics: educational level, employment status, and income. These are all standard characteristics of socioeconomic status which may elucidate different aspects of inequity. All characteristics were assessed in 2010 and 2017, respectively.

Educational level was defined as the highest completed level of education for each individual. Information on educational level was obtained from the Population Education Register, which is a nationwide register with information on highest completed educational level and year of completed education [21]. Educations are classified according to the ISCED2011 (International Standard Classification of Education) [22]. For this study, educational level was categorised into three groups: elementary (preliminary, primary, and lower secondary; ISCED levels 1–2; ≤ 9 years), short (upper secondary and postsecondary; ISCED levels 3–4, 10–12 years), and medium/long (tertiary education; ISCED levels 5–8, ≥ 13 years).

Employment status was defined based on information about labour market affiliation. This information was obtained from the Employment Classification Module, which is a nationwide register with annual information on the most important source of an individual’s income [23]. Employment status was categorised info five groups: employment, unemployment, disability pension, early retirement, and retirement.

Income was defined as equivalised disposable household income for each individual. Information on income was obtained from the Income Statistics Register, which is a nationwide register with information on annual income, tax, and wages [24]. Income was categorised into four groups based on quartiles: low (lowest 25% quartile), low-medium, medium–high, high (highest 25% quartile) within calendar year and age groups.

### Health status as a proxy of need for chiropractic care

Musculoskeletal conditions are the main reason for seeking chiropractic care [3, 5]. Therefore, measures of health status in relation to chiropractic care were chosen with a specific focus on musculoskeletal conditions. Measures of health status included demographics, poor self-rated physical health, activity limitations, musculoskeletal pain, number of musculoskeletal conditions (only those of relevance for chiropractic consultation), and number of chronic diseases (excluding those included as musculoskeletal conditions).

Demographics included age (30–44, 45–64, 65–74, ≥ 75 years by January 1, in 2010 and 2017, respectively) and sex (female, male).

Poor self-rated physical health was defined based on a question in DNHS: “How do you think your health is, all in all?” with the following answer options: “Excellent”, “Very good”, “Good”, “Less good”, and “Poor”. Poor self-rated health was a binary variable defined by having answered “Less good”, or “Poor”.

Activity limitations was defined based two questions in DNHS: “The following questions are about activities in everyday life. Are you restricted in these activities because of your health? If so, how much?”: “Lighter activities, such as moving a table, vacuuming or biking” and “Walk several floors up stairs?” with the following answer options: “Yes, very limited”, “Yes, a bit limited”, and “No, not at all”. Activity limitations was a binary variable defined by having answered “Yes, very limited” to at least one of the questions.

Musculoskeletal pain was defined based on three questions in DNHS: “Have you been bothered by any of the pain and discomfort mentioned here in the past 14 days? Were you very or a little bothered?”: “Pain and discomfort in the shoulder or neck?”, “Pain and discomfort in the arms, hands, legs, knees, hips or joints?” and “Pain and discomfort in the back or lower back” with the following answer options: “Yes, very bothered”, “Yes, a bit bothered”, and “No”. Musculoskeletal pain was a binary variable defined by having answered “Yes, very bothered” to at least one of the questions.

Number of musculoskeletal conditions and chronic diseases, respectively, were defined based on information from the Danish National Patient Register, the Danish National Prescription Register and DNHS. The Danish National Patient Register is a nationwide register with information about all hospital contacts, including date of admission and discharge, and diagnoses [25]. Diagnoses are classified according to International Statistical Classification of Diseases and Related Health Problems (ICD). The Danish National Prescription Register is a nationwide register with information about all prescription redemptions in Danish community pharmacies, including date of redemption, drug dispensed, and drug user [26]. Drugs are classified according to Anatomic Therapeutic Chemical classification (ATC) code.

Musculoskeletal conditions included osteoarthritis, spondylopathies and other dorsopathies, and fibromyalgia. The ICD codes used to identify these conditions in the Danish National Patient Register are listed in Supplementary Table S1 (see Additional file 1). Rheumatoid arthritis and osteoporosis were not included as musculoskeletal conditions for purposes of this analysis. We chose to designate these conditions as chronic diseases for this analysis because chiropractors most commonly treat musculoskeletal conditions with spinal manipulation and there is no current evidence to suggest disease modification of these conditions with spinal manipulation. Number of musculoskeletal conditions was defined by a count of diagnosed conditions identified in the registers for each individual complemented with self-reported information obtained from DNHS. DNHS included information on osteoarthritis, and herniated disc or other spinal diseases. Furthermore, individuals reporting rheumatoid arthritis in DNHS without having a recorded diagnosis of rheumatoid arthritis were considered suffering from osteoarthritis and not rheumatoid arthritis. This was done since the self-reported information on rheumatoid arthritis in DNHS has a positive predictive value of only 13% [27].

The 44 diagnoses included as chronic disease are listed in Supplementary Table S2 (see Additional file 1) with ICD and ATC codes for definition. The list was based on a definition of multimorbidity [28], with exclusion of the diagnoses included as musculoskeletal conditions described above. Number of chronic diseases was defined by a count of diagnosed diseases identified in the registers for each individual complemented with self-reported information obtained from DNHS. DNHS included information on asthma, allergy, diabetes, hypertension, myocardial infarction, angina pectoris, stroke, chronic bronchitis, hyperinflated lungs, COPD (Chronic obstructive pulmonary disease), osteoporosis, cancer, migraine or headache, mental disorders, cataracts, and tinnitus.

### Statistical analysis

A descriptive analysis of the two study populations was performed by means of frequencies (N, %) stratified by sex and calendar year.

Social inequity in chiropractic utilisation was examined using logistic regression with chiropractic utilisation (yes, no) as the outcome. Separate analyses were performed for educational level, employment status, and income, respectively. This was done to avoid the so-called “Table 2 Fallacy” [29] where interpretations of effect estimates from joint analyses are dissimilar. Analyses were adjusted for health status, including age, and stratified by sex and year. Stratification by sex was done to accommodate potential differences in social inequity between men and women. Previous studies found sex differences in chiropractic utilisation [3], and men and women may potentially act differently depending on their socioeconomic status. To limit bias due to different sampling probabilities and differential non-response, we applied survey weights calculated by Statistics Denmark for individuals in each survey based on information such as sex, age, education, and income [30, 31]. Social inequity in chiropractic utilisation was presented as an odds ratio (OR) with corresponding 95% confidence interval (CI).

To quantify the degree of social inequity in chiropractic utilisation for each of the three characteristics of socioeconomic status we estimated the concentration index of inequity with 95% confidence interval [32, 33]. The index is a measure of the extent of inequities in health that is systematically associated with socioeconomic status. The concentration index takes values from -1 to 1, where 0 indicates no inequality. A positive value indicates health inequity in favour of individuals with higher socioeconomic status, whereas a negative value indicates health inequity in favour of individuals with lower socioeconomic status.

When estimating the concentration index related to a certain characteristic of socioeconomic status, ranking the groups within the characteristic is required. Therefore, employment status was reduced to three groups: employment, unemployment, and outside the labour market (including disability pension, early retirement, and retirement), and only assessed for individuals aged 30–64 years. The latter was done since socioeconomic ranking of individuals above retirement age (65 years) cannot be based on employment status. There is a natural ranking within educational level and income, but for comparison the concentration indexes for these characteristics were also assessed for individuals aged 30–64 years.

The concentration index was estimated in a regression analysis adjusting for health status and stratified by sex and year. In this analysis, health status included activity limitations, musculoskeletal pain, and number of musculoskeletal conditions. Other measures of health status (i.e., age, poor self-rated physical health, and number of chronic diseases) had to be excluded to avoid empty cells in the analysis.

Changes in social inequity between 2010 and 2017 stratified by sex was tested by including an interaction with year when estimating the concentration index.

## Results

**Fig. 1:**
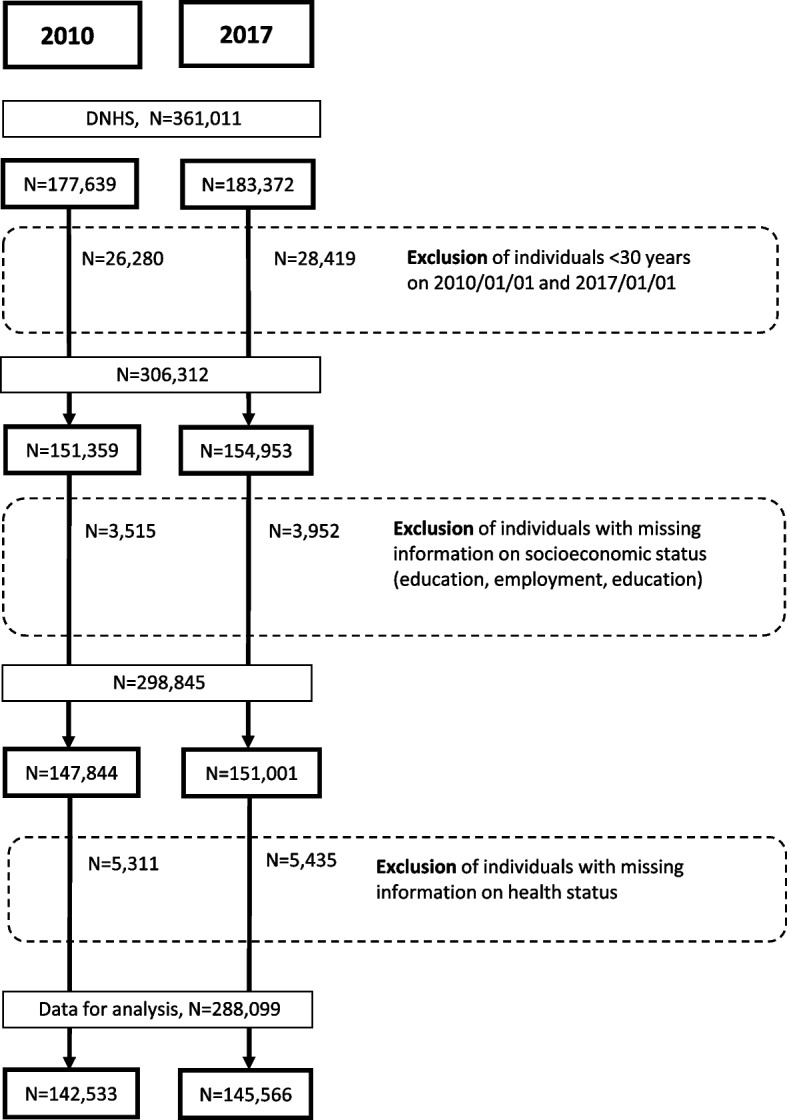
Data flow diagram

We identified a total of 361,011 individuals who participated in the Danish National Health Survey in 2010 or 2017. After exclusion of individuals below the age of 30 years on 2010/01/01 and 2017/01/01, respectively, as well as individuals with missing information on socioeconomic status or health status, we arrived at a final study population of 288,099 individuals (Fig. 1).

### Baseline characteristics

In general, baseline characteristics are similar between the two years when comparing between study populations and sexes, however, there are minor differences (Table 1). The 2017 population was slightly older than the 2010 population, a larger proportion had a long education, and a larger proportion was on retirement in 2017. Furthermore, a larger proportion of the 2017 population reported poor self-rated health, activity limitations, and musculoskeletal pain, and the 2017 population had more musculoskeletal conditions and chronic diseases. In both years, a larger proportion of males were employed compared with females. Conversely, a larger proportion of females were early retired or unemployed. Furthermore, a larger proportion of females reported poor self-rated physical health, activity limitations, musculoskeletal pain and had musculoskeletal conditions compared with males.

**Table 1:** Baseline characteristics of the two study populations stratified by sex. Values are number (N) and percentage (%)

	Female		Male	
	2010	2017	2010	2017
Age group				
30–44 years	21,625 (28.3%)	17,809 (22.8%)	16,933 (25.6%)	13,760 (20.4%)
45–64 years	34,713 (45.4%)	34,445 (44.1%)	30,930 (46.8%)	29,539 (43.8%)
65–74 years	12,911 (16.9%)	16,392 (21.0%)	12,235 (18.5%)	15,669 (23.2%)
≥ 75 years	7,186 (9.4%)	9,499 (12.2%)	6,000 (9.1%)	8,453 (12.5%)
Education				
Elementary (≤ 9 years)	21,153 (27.7%)	17,623 (22.6%)	15,378 (23.3%)	13,822 (20.5%)
Short (10–12 years)	29,934 (39.2%)	30,900 (39.5%)	31,580 (47.8%)	32,060 (47.6%)
Medium/long (≥ 13 years)	25,348 (33.2%)	29,622 (37.9%)	19,140 (29.0%)	21,539 (32.0%)
Employment				
Retirement	20,037 (26.2%)	25,231 (32.3%)	16,948 (25.6%)	21,899 (32.5%)
Early retirement	4,161 (5.4%)	2,209 (2.8%)	3,427 (5.2%)	1,336 (2.0%)
Disability pension	3,914 (5.1%)	3,286 (4.2%)	2,376 (3.6%)	1,980 (2.9%)
Unemployment	3,888 (5.1%)	5,277 (6.8%)	2,957 (4.5%)	2,958 (4.4%)
Employment	44,435 (58.1%)	42,142 (53.9%)	40,390 (61.1%)	39,248 (58.2%)
Income				
Low	15,219 (19.9%)	15,724 (20.1%)	11,224 (17.0%)	11,131 (16.5%)
Low-medium	19,078 (25.0%)	20,024 (25.6%)	15,757 (23.8%)	16,311 (24.2%)
Medium–high	20,602 (27.0%)	21,189 (27.1%)	18,688 (28.3%)	19,212 (28.5%)
High	21,536 (28.2%)	21,208 (27.1%)	20,429 (30.9%)	20,767 (30.8%)
Poor self-rated physical health				
No	63,715 (83.4%)	63,471 (81.2%)	56,854 (86.0%)	56,564 (83.9%)
Yes	12,720 (16.6%)	14,674 (18.8%)	9,244 (14.0%)	10,857 (16.1%)
Activity limitations				
No	68,169 (89.2%)	68,658 (87.9%)	61,142 (92.5%)	61,466 (91.2%)
Yes	8,266 (10.8%)	9,487 (12.1%)	4,956 (7.5%)	5,955 (8.8%)
Musculoskeletal pain				
No	53,997 (70.6%)	52,342 (67.0%)	51,716 (78.2%)	50,236 (74.5%)
Yes	22,438 (29.4%)	25,803 (33.0%)	14,382 (21.8%)	17,185 (25.5%)
Number of musculoskeletal conditions				
0	47,141 (61.7%)	44,319 (56.7%)	43,274 (65.5%)	41,579 (61.7%)
1	21,832 (28.6%)	24,605 (31.5%)	17,460 (26.4%)	19,160 (28.4%)
≥ 2	7,462 (9.8%)	9,221 (11.8%)	5,364 (8,1%)	6,682 (9.9%)
Number of chronic diseases^a^				
0	19,020 (24.9%)	16,424 (21.0%)	20,737 (31.4%)	17,450 (25.9%)
1	18,125 (23.7%)	17,372 (22.2%)	15,675 (23.7%)	15,226 (22.6%)
2	13,978 (18.3%)	14,173 (18.1%)	10,212 (15.5%)	10,797 (16.0%)
3	9,927 (13.0%)	10,877 (13.9%)	7,450 (11.3%)	8,419 (12.5%)
≥ 4	15,385 (20.1%)	19,299 (24.7%)	12,024 (18.2%)	15,529 (23.0%)

^a^Excluding those included as musculoskeletal conditions

### Social inequity in chiropractic utilisation

**Fig. 2:**
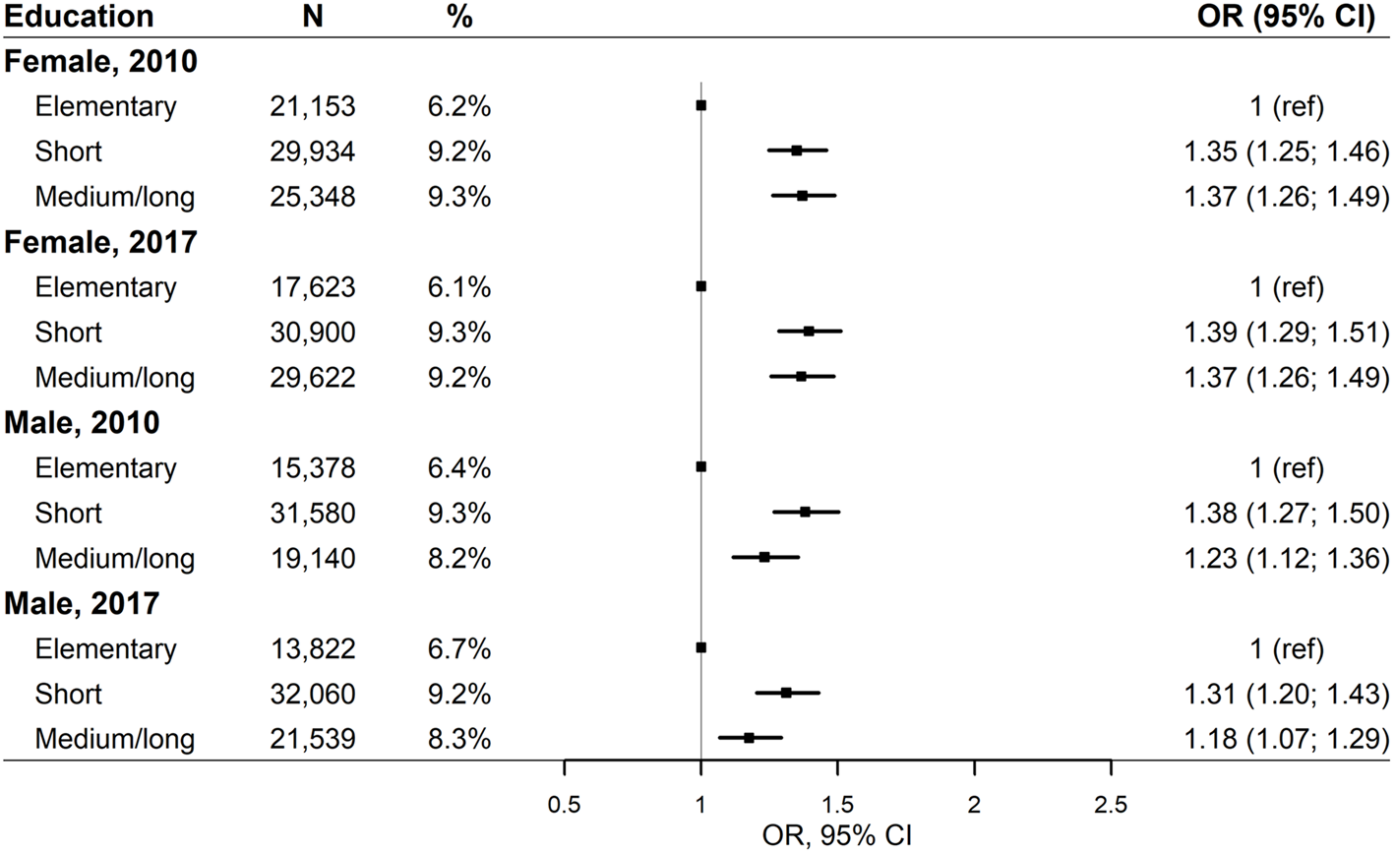
Educational level and chiropractic utilisation adjusted for health status, including age, and stratified by sex and year

In 2010, we found significantly higher odds of chiropractic utilisation among females with short education (OR = 1.35; 95% CI: 1.25; 1.46) and medium/long education (OR = 1.37; 95% CI: 1.26;1.49) compared with females with elementary education (Fig. 2). Similarly, we found significantly higher odds of chiropractic utilisation among males with short education (OR = 1.38; 95% CI: 1.27;1.50) and medium/long education (OR = 1.23; 95% CI 1.12;1.36) compared with males with elementary education in 2010. Similar inequities were found among females and males in 2017.

**Fig. 3:**
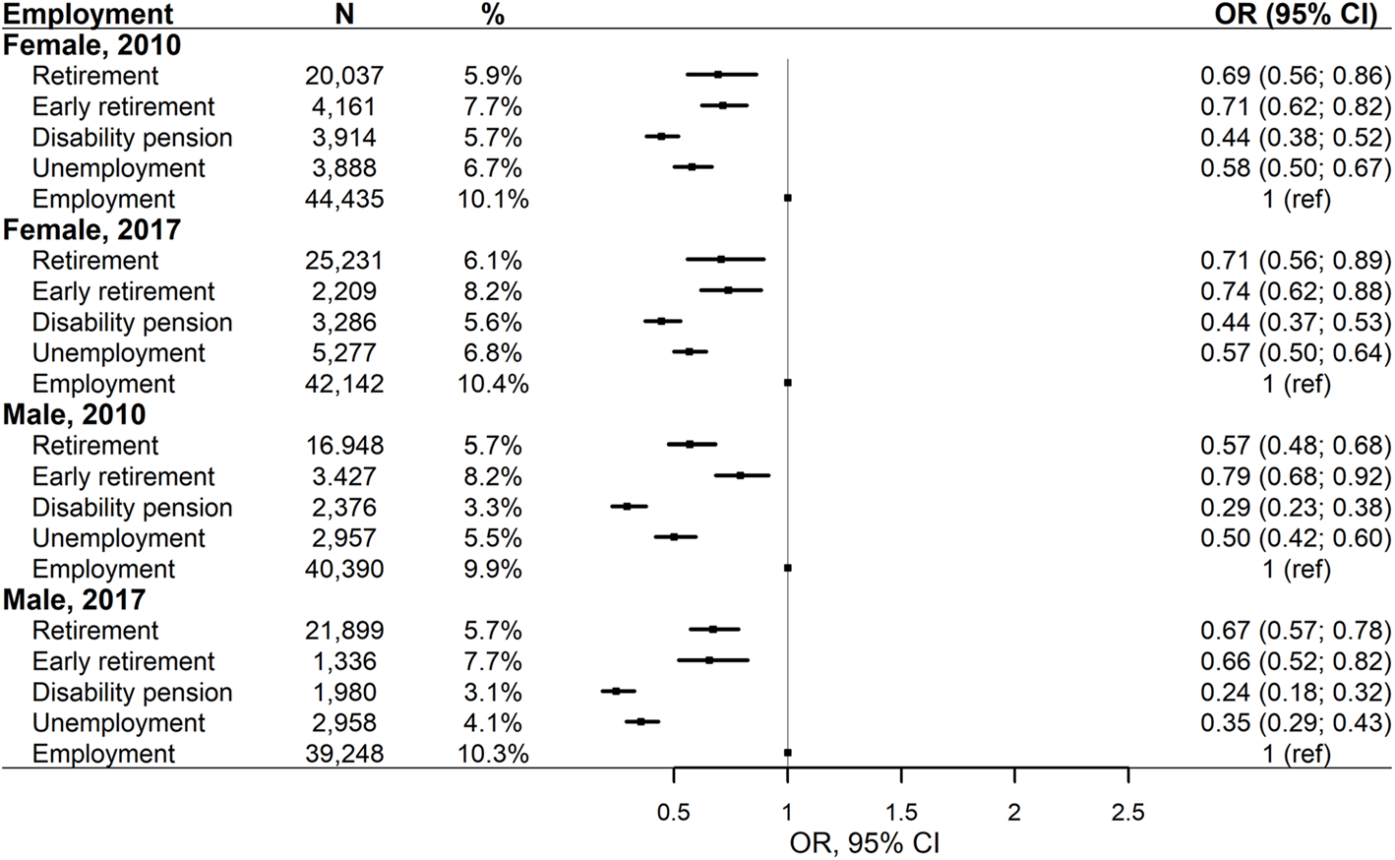
Employment status and chiropractic utilisation adjusted for health status, including age, and stratified by sex and year

In general, the odds of chiropractic utilisation were higher among individuals with employment than among individuals without employment. In 2010, we found significantly lower odds of chiropractic utilisation among retired females (OR = 0.69, 95% CI: 0.56;0.86), early retired females (OR = 0.71, 95% CI: 0.62;0.82), females receiving disability pension (OR = 0.44, 95% CI: 0.38;0.52), and unemployed females (OR = 0.58, 95% CI: 0.50;0.67) compared with employed females (Fig. 3). Similarly, we found significantly lower odds of chiropractic utilisation among retired males (OR = 0.57, 95% CI: 0.48;0.68), early retired males (OR = 0.79, 95% CI: 0.68;0.92), males receiving disability pension (OR = 0.29, 95% CI: 0.23;0.38), and unemployed males (OR = 0.50, 95% CI: 0.42;0.60) compared with employed males. Similar inequities were found among females and males in 2017.

**Fig. 4:**
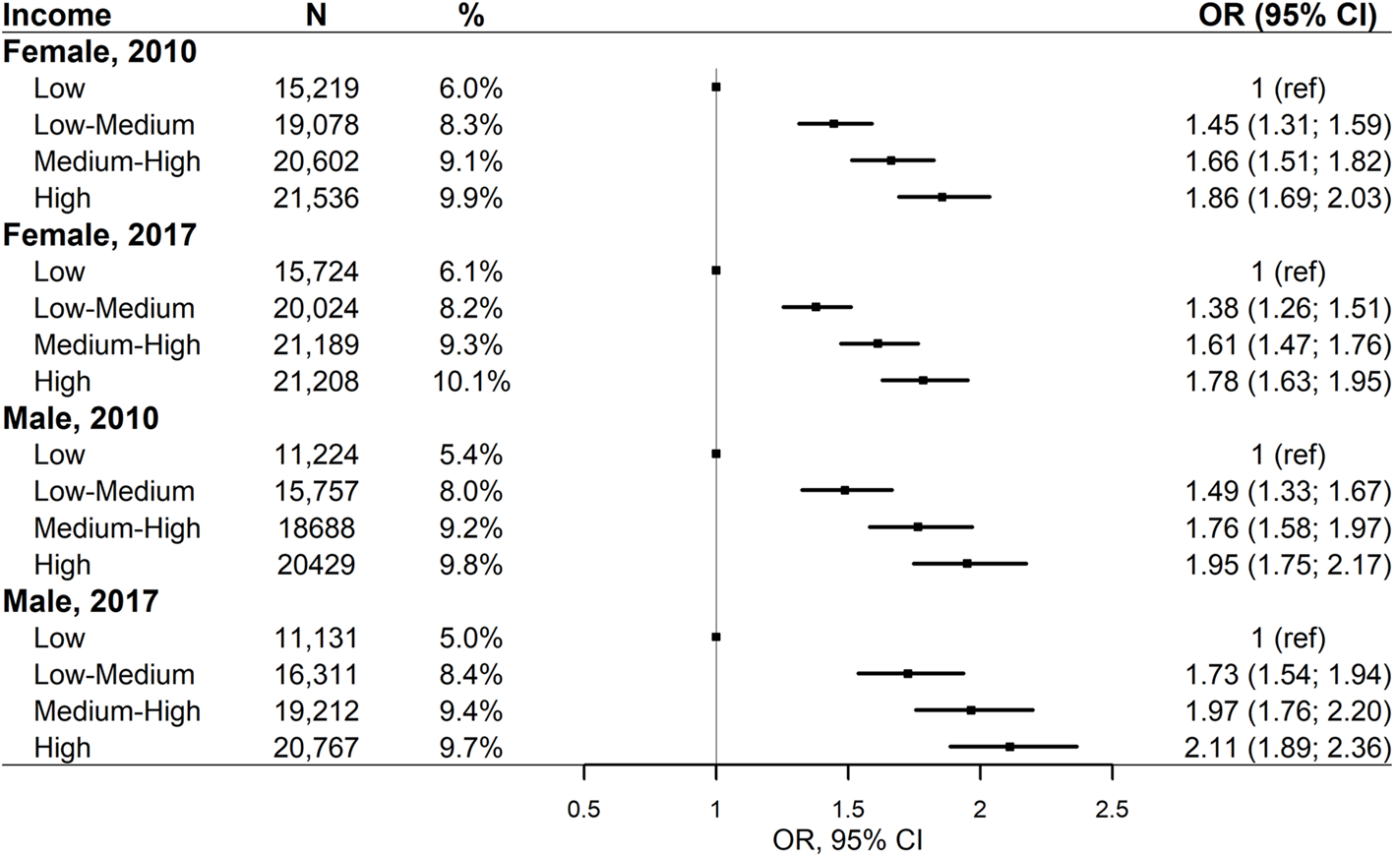
Income and chiropractic utilisation adjusted for health status, including age, and stratified by sex and year

In general, the odds of chiropractic utilisation increased with higher income (Fig. 4). In 2010, we found significantly higher chiropractic utilisation among females with low-medium (OR = 1.45; 95% CI: 1.31;1.59), medium–high (OR = 1.66; 95% CI: 1.51;1.82), and high income (OR = 1.86; 95% CI: 1.69;2.03) compared with females with low income. Similarly, we found significantly higher chiropractic utilisation among males with low-medium (OR = 1.49; 95% CI: 1.33;1.67), medium–high (OR = 1.76; 95% CI: 1.58;1.97), and high income (OR = 1.95; 95% CI: 1.75;2.17) compared with males with low income in 2010. Similar inequities were found among females and males in 2017.

### Concentration index of inequity in chiropractic utilisation

**Fig. 5:**
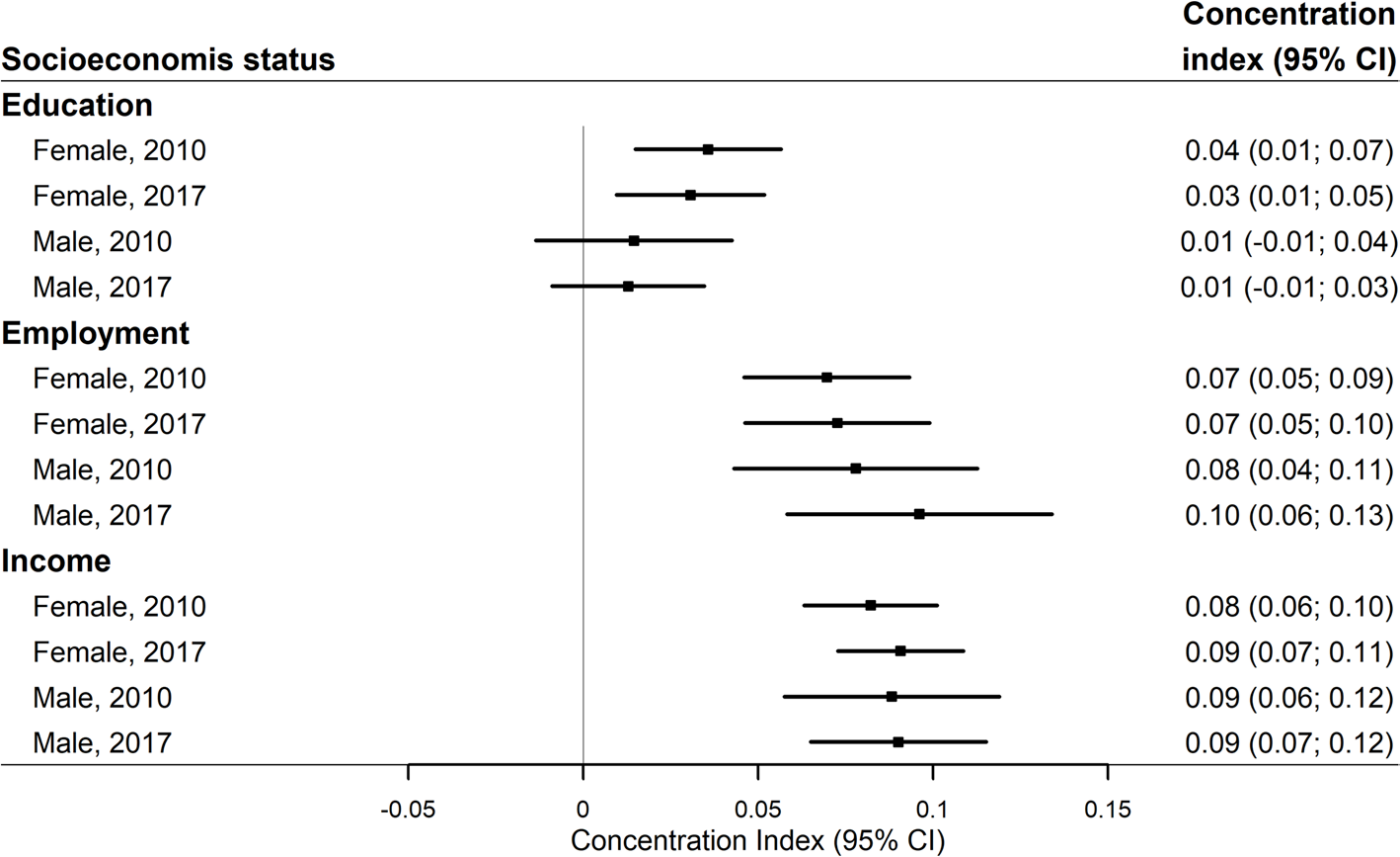
Concentration index of inequity in chiropractic utilisation by educational level, employment status and income among individuals aged 30 to 64 years, stratified by sex and year

The concentration index indicates inequity in chiropractic utilisation in favour of individuals with higher socioeconomic status since all index values are positive, although the confidence interval of the index related to education includes zero for males in both 2010 and 2017 (Fig. 5). Income and employment status contributed to a larger degree to inequity compared with educational level. There was no difference in the inequity between 2010 and 2017 for the three socioeconomic characteristics, stratified by sex (*p*-values ranging between 0.47 and 0.92).

## Discussion

The main finding of this study is the presence of social inequity in chiropractic utilisation in the general Danish population when adjusting for health status. The results showed that a significantly lower proportion of individuals with elementary education utilised chiropractic care compared with individuals with higher educational level. Similarly, a significantly lower proportion of individuals with the lowest income (lowest quartile of equalised disposable household income) utilised chiropractic care compared with individuals with higher income levels. A significantly larger proportion of employed individuals utilised chiropractic care compared with individuals who were unemployed, receiving disability pension or retired. Inequity between groups characterised by income or employment status was more pronounced than inequity between groups characterised by educational level. The social inequity did not change between 2010 and 2017.

We have not identified any previous studies that aimed for assessing social inequity in chiropractic utilisation in a general population adjusted for health status as a proxy of need for chiropractic care. Some studies asses utilisation in specific populations with specific needs, e.g., individuals with chronic back disorders [34], whereas other studies report crude measures of utilisation in different socioeconomic groups, e.g., within different levels of education and employment [35]. A study on determinants of variability in chiropractic use in the United States adult population evaluated sex, ethnicity, income, and education, but also health indicators (e.g., arthritis) and perceived health status [36]. When all factors were mutually adjusted, the authors found significantly higher chiropractic utilisation among individuals with higher family income. No significant association between chiropractic utilisation and education was found. The latter is in contrast to the present study in which we found a significant inequity in chiropractic utilisation due to education and income, although the income-related inequity was larger. In the present study, however, education and income were not mutually adjusted which may explain the discrepancy in results between the two studies.

An Australian study found significantly higher chiropractic utilisation among individuals with higher income when adjusting for having visited a medical doctor in the past 12 months and having visited a medical doctor for back problems [37]. The study found no association between chiropractic utilisation and education (post-secondary yes/no) or employment (employed/unemployed) in univariable analyses, i.e., with no adjustment for other factors.

An American study adjusted for health insurance status and perceived health status (as well as other factors) when assessing the association between chiropractic utilisation and education or income (mutually adjusted) in young adults aged 18 to 27 years [38], and found no association with chiropractic utilisation in this young population. The present study included adults aged 30 years or older.

Considering utilisation of health services other than chiropractic, previous studies have shown social inequity in utilisation of specialist physicians [39, 40] and hospital outpatient care [39] in Norway, another Scandinavian country with a universal healthcare system, but with copayment for health services other than public hospital inpatient care. An international review found socioeconomic inequalities in utilisation to be more prevalent among specialists than among primary-care physicians [41]. Thus, social inequity in utilisation is not limited to chiropractic care, and the underlying drivers seem to affect utilisation of multiple healthcare services within various healthcare systems.

Although correlated, each of the three characteristics of socioeconomic status considered in this study may represent different aspects of social inequity. For example, inequity related to educational level may indicate an effect of heath literacy on chiropractic utilisation, whereas the observed income-related inequity may indicate that private co-payment affects utilisation between socioeconomic groups. Employment is correlated with income, but the observed employment-related inequity may also reflect inequity caused by differences in access to private health insurance, since most private health insurances were obtained as part of a job agreement [9]. A previous Danish study found a positive effect of private health insurance on chiropractic utilisation [42]. In this study, we did not have information on private health insurance status.

This population-based study has several strengths including a large, representative sample of the general population and high-quality information from nationwide registers. This limits the impact of selection bias, limits the uncertainty of the estimated inequities, and ensures a high level of validity. The registers used in the present study are reliable and validated with a high degree of completeness and validity [19–21, 23–25].

The study included a representative sample of individuals in the general population which increased the generalisability of the findings compared with studies on a population of individuals with specific symptoms, disorders, or diagnoses. We performed weighted analyses to limit bias due to different sampling probabilities and differential non-response. Social inequity was presented using both a relative measure as the OR using logistic regression and the concentration index.

In the study, we adjusted for several variables quantifying the individuals’ health status to ensure assessment of social inequity in chiropractic utilisation beyond differences in health status. Health status was used as a proxy of need for chiropractic care, and there is no standard definition of variables to include to quantify need for chiropractic care. We have included what we consider as relevant diseases, symptoms, and conditions by using both self-reported information and information from hospital contacts, and the inclusion of several variables quantifying health status (self-rated physical health, activity limitations, musculoskeletal pain, number of musculoskeletal conditions, and number of chronic diseases) is a strength of the study. Musculoskeletal pain, which appears as a highly relevant measure in relation to need for chiropractic care, is not commonly included in other studies of chiropractic utilisation. Perceived need of care is an important aspect of health care needs, and self-rated physical health was included to reflect the individual’s own perception of their health and in turn their perceived need of care. Other elements such as e.g. health literacy may be considered as part of the social inequity we want to assess and thus should not be adjusted for.

The main limitation of the study is that health status may change during the time period in which chiropractic utilisation is assessed. Most information on health status was obtained from the DNHS which was completed in the beginning of a year, whereas chiropractic utilisation was assessed during the whole year. Thus, we may underestimate an individuals’ need for chiropractic care. Also, few chiropractors practice outside of the official Danish health insurance system, and we do not have utilisation data from those chiropractors. It is, however, unlikely that inclusion of data from these chiropractors would affect the results of the study.

The results of the present study may serve as essential input to healthcare planners and inform strategies to ensure equal treatment for equal need, regardless of socioeconomic position. Potential actions could include a reconsideration of the out-of-pocket expense for patients, as well as initiatives to inspire increased awareness and support targeted vulnerable patient groups. Future research should investigate the role of private health insurances and how they influence social inequity in chiropractic utilisation. Furthermore, other aspects of utilisation could be investigated, including for example geographical differences in access to chiropractic and their influence on chiropractic utilisation.

## Conclusion

This study demonstrated social inequity in chiropractic utilisation in Denmark beyond differences in health status as a proxy of need for chiropractic care in the general population, where income- and employment-related inequity was found to be more pronounced than inequity related to educational level. Using a detailed quantification of health status, the study presented a novel approach to assessing differences in chiropractic utilisation between socioeconomic groups, and the results suggest that new strategies are required if equal treatment for equal need is the goal.

### Supplementary Information

Supplementary Material 1.

## Data Availability

Data underlying this study cannot be shared publicly due to data privacy regulations by Statistics Denmark.

## Abbreviations

DNHS: Danish National Health Survey
OR: Odds ratio
CI: Confidence interval
