# Supplementary material for: Social inequity in chiropractic utilisation – a cross-sectional study in Denmark, 2010 and 2017
Source: Chiropr Man Therap. 2024 Jul 15;32:27. doi: 10.1186/s12998-024-00548-x (PMC11251339; doi:10.1186/s12998-024-00548-x)
Supplement: Supplementary file 1 — Supplementary Material 1. [file 12998_2024_548_MOESM1_ESM.docx]

Table S1. Diagnoses included as musculoskeletal conditions. Hospital contacts within 10 years prior to the study year (i.e., 2010 or 2017) were included.

| **Disease** | **ICD-10 codes^*^** |
| --- | --- |
| Osteoarthritis | **M15**  **M16**  **M17**  **M18**  **M19** |
| Spondylopathies and other dorsopathies | **M40**  **M41**  **M42**  **M43**  **M45**  **M46**  **M47**  **M48**  **M49**  **M50**  **M51**  **M53**  **M54** |
| Fibromyalgia | **M79.7** |

^*^Including all subcodes

Table S2. Diagnoses included as chronic disease. Hospital contacts within the given inclusion time prior to the study year (i.e., 2010 or 2017) were included. Medicine prescriptions were included if minimum two prescriptions were redeemed within two years prior to the study year. The definition of chronic disease was based on the approach used by Moller et al. 2020^§^.

| **Disease** | **Inclusion time**  **(years)** | **ICD-10 codes^*^** | **ATC codes and indication codes** |
| --- | --- | --- | --- |
| Diabetes | 10 | **E10**  **E11** | **A10A (**not A10AE56)  **A10B** (not A10BJ02) |
| Thyroid diseases | 5 | **E00**  **E01**  **E02**  **E03**  **E04**  **E05**  **E06**  **E07** | **H03** |
| Ischemic heart disease and heart failure | 5 | **I20**  **I21**  **I23**  **I24**  **I25**  **I50**  **I11**  **I13** | **CO1A**  **CO1B**  **CO1D**  **CO1E**  **C01AA05**  **C09A** (only with indication code 430) |
| Pulmonary heart disease and diseases of pulmonary circulation | 5 | **I26**  **I27**  **I28** |  |
| Atrial fabrillation and flutter | 10 | **I48** |  |
| Aortic and mitral valve disease | 5 | **I05**  **I06**  **I34**  **I35** |  |
| Atherosclerosis | 10 | **I70** |  |
| Phlebitis and thrombophlebitis | 5 | **I80** |  |
| Hypertensive diseases | 5 | **I10**  **I12**  **I15** | **C02A**  **C02B**  **C02C**  **C02DA**  **C02L**  **C03A**  **C03B**  **C03D**  **C03E**  **C03X**  **C07C**  **C07D**  **C08G**  **C09BA**  **C09DA**  **C09XA52**  **C02DB**  **C02DD**  **C02DG**  **C07**  **C07F**  **C08**  **C09BB**  **C09DB**  **C09** |
| Disorders of lipoprotein metabolism and other lipidaemias | 10 | **E78.0**  **E78.2**  **E78.4**  **E78.5** | **C10** |
| Crohns’s disease and ulcerative colitis | 10 | **K50**  **K51** |  |
| Irritable bowel disease | 10 | **K58** |  |
| Diseases of liver, biliary tract, and pancreas | 10 | **K71**  **K72**  **K73**  **K74**  **K75**  **K76**  **K77**  **K86.1**  **K87** |  |
| Stroke and transient cerebral ischemic attacks and related syndromes and vascular syndromes of the brain in cerebrovascular diseases | 10 | **G45**  **G46**  **I60**  **I61**  **I62**  **I63**  **I64**  **I65**  **I66**  **I67**  **I68**  **I69** |  |
| Epilepsy | 5 | **G40**  **G41** | **N03**  **N05BA** (only with indication code 155 or 753)  **N05CD** (only with indication code 155 or 753) |
| Migraine and other headache syndromes | 10 | **G43**  **G44** | **N02C** |
| Dementia | 10 | **F00**  **F01**  **F02**  **F03**  **G30**  **G31.8B**  **G31.8E**  **G31.9**  **G31.0B** | **N06D** (only with indication code 329, 330, or 331) |
| Parkinson’s disease | 10 | **G20**  **G21**  **G22**  **F02.3** | **N04** |
| Sclerosis | 10 | **G35** |  |
| COPD and chronic lower respiratory diseases | 10 | **J40**  **J41**  **J42**  **J43**  **J44**  **J47**  **J96** | **R03AC18**  **R03AC19**  **R03AL02**  **R03AL03**  **R03AL04**  **R03AL05**  **R03AL06** **R03AL09**  **R03BB04**  **R03BB05**  **R03BB06**  **R03BB07**  **R03DX07**  (all ATC-codes only with indication code 379 or 464) |
| Asthma | 10 | **J45** | **R03DC03** (only with indication code 202, 203, or 822) |
| Chronic kidney disease | 10 | **N18** |  |
| Malignant neoplasms of digestive organs | 10 | **C15-C26** |  |
| Malignant neoplasms of respiratory and intrathoracic organs | 10 | **C30-C39** |  |
| Malignant melanoma of skin | 10 | **C43** |  |
| Malignant neoplasm of breast | 10 | **C50** |  |
| Malignant neoplasms of genital organs | 10 | **C51-C58**  **C60-C63** |  |
| Other malignant neoplasms excluding metastases | 10 | **C00-C14**  **C40-C41**  **C45-C49**  **C64-C68**  **C69-C72**  **C73-C75**  **C81-C96** |  |
| Depression | 5 | **F32**  **F33**  **F34.1**  **F06.32** | **N06A** (only with indication code 168) |
| Anxiety | 5 | **F40.1**  **F41.1** | All prescriptions with indication code 163 or 371 |
| Schizophrenia | 10 | **F20**  **F21**  **F22**  **F25**  **F28**  **F29** | **N05AX13 N05AX12 N05AH03 N05AX08** |
| Bipolar affective disorder | 10 | **F30**  **F31** | **N05A** (only with indication code 461 or 631)  **N06A** (only with indication code 461 or 631) |
| PTSD | 5 | **F43.1** |  |
| Obsessive-compulsive disorder | 10 | **F42** | **N06A** (only with indication code 472 or 596) |
| Eating disorders | 10 | **F50** |  |
| Alcohol attributable diseases | 10 | **G31.2**  **G62.1**  **G72.1**  **K29.2**  **K70**  **K86.0** |  |
| Respiratory allergy | 10 | **J30.1**  **J30.2**  **J30.3**  **J30.4** | **V01AA02**  **V01AA03**  **V01AA05**  **V01AA11**  **R01AC**  **R01AD**  **R06A**  **S01G**  **R01BA52** |
| HIV/AIDS | 10 | **B20**  **B21**  **B22**  **B23**  **B24** |  |
| Injuries of nerves and spinal cord and paralytic syndromes | 10 | **G81**  **G82**  **G83**  **S14**  **S24**  **S34**  **T09.3** |  |
| Blindness | 10 | **H54** |  |
| Tinnitus | 10 | **H93.1** |  |
| Congenital malformations, deformations and chromosomal abnormalities | 10 | **Q00-Q99** |  |
| Osteoporosis | 10 | **M80**  **M81**  **M82** | **M05BA01**  **M05BA04**  **M05BA06** (only 150 mg tablets):  **M05BA07**  **M05BB01**  **M05BB03**  **M05BX03**  **G03XC01**  **H05AA02**  **H05AA03** |
| Inflammatory polyarthropathies | 10 | **M05**  **M06.0**  **M06.8**  **M07.0**  **M07.1**  **M07.3**  **M10.0**  **M10.9** | All prescriptions with indication code 147, 402 or 461 |

^§^ Moller, S.P., et al., *Patterns of multimorbidity and demographic profile of latent classes in a Danish population—A register-based study.* PloS one, 2020. 15(8): p. e0237375-e0237375.

^*^Including all subcodes
